# Best Practices of Hospitals in Management of Epidemic Conditions: A Scoping Review

**DOI:** 10.31661/gmj.v12i.2824

**Published:** 2023-08-20

**Authors:** Ali Tahmasebi, Iravan Masoudi Asl, Aidin Aryankhesal, Soudabeh Vatankhah, Gholamreza Masoumi

**Affiliations:** ^1^ Department of Health Services Management, School of Health Management and Information Sciences, Iran University of Medical Sciences, Tehran, Iran.; ^2^ Department of Health in Disaster and Emergencies, School of Health Management and Information Sciences, Iran University of Medical Sciences, Tehran, Iran.

**Keywords:** Practice, Hospital, Management, Epidemic Condition

## Abstract

Background: Many hospitals globally have valuable experiences in preparing for management and responding to infectious epidemics. Identifying and analyzing these experiences can provide comprehensive and practical data for decision-making and effective performance. This study aimed to conduct a scoping review and content analysis of the best practices of hospital (private or public) management in epidemic conditions.Materials and Methods: This research is a scoping review and content analysis, conducted in 2021. Data was collected by searching different databases, including Pubmed, Scopus, Web of Sciences, ProQuest, websites, search engines, and public reports without time limits. Content analysis was performed for data analysis.Results: We retrieved 8842 records from databases and other sources. Finally, 24 studies from 12 countries were selected for analysis. Most studies belonged to the United States (9 cases), and most subjects were on Coronavirus disease 2019 (Covid-19) (19 studies). We classified the results into two major categories of in-hospital executive readiness and logistic readiness. Executive readiness included 11 main categories (physical structure, resource management, exposure reduction, patients and caregivers’ management, corpse management, disinfection, staff support, patient admission, instructions and guidelines, tele- communication, and education) and 26 sub-categories. Logistic readiness consisted of three major categories (leadership/team making, communication, and using capabilities) and five sub-categories.Conclusion: Healthcare managers can use the identified categories and dimensions of managerial readiness and responsiveness as an action plan during an infectious disease epidemic.

## Introduction

Due to climate and environmental changes and other known and unknown events in recent decades, a new diseases called "Emerging Diseases" are threatening global health. The main reasons behind the emergence and prevalence of these diseases are facilitated international traveling, changes in diet and lifestyle, migration, marginalization, and others. Thus, environmental changes can lead to new infectious or communicable diseases [1]. Regarding the scale and effects of infectious epidemics, health systems must have plans to deal with these situations without planning and strong health policies, countries will face many challenges imposing a high workload on the healthcare system [2].

These issues include economic turmoil, social anxiety, unresponsiveness of the healthcare toward patients, and loss of health professionals and healthcare providers. Therefore, the health system needs to address these diseases in a precise and targeted manner [2].

An infectious disease outbreak causes many challenges for health centers, especially hospitals and healthcare providers. Healthcare staff compliance with guidelines on preventing and control of infection, isolating respiratory patients, sanitizing environment and equipment, absence of obligatory training, isolation rooms, waiting rooms and high-quality equipment, minimizing crowds, immediate identification of infected people, minimizing visitors, and easy access to hand hygiene facilities are among the most important challenges [3]. A qualitative study by Kian Lio et al. in Hubei, China, with the participation of nurses and physicians, showed that physical problems including exhaustion due to work pressure, physical and psychological disorders of the staff, lack of isolated rooms, lack of medical staff, different and contradictory guidelines, and staff communication-related problems with different specialties and experiences were the most common problems in hospitals [4]. Other studies showed that the lack of medical staff, lack of Personal Protective Equipment (PPE), staff exhaustion and heavy workload, staff mental problems, unknown nature of the disease, hospitals economic challenges, and communication-related problems were other challenges in hospitals in Coronavirus disease 2019 (Covid-19) patient management [3][4][5][6][7][8]. The review of literature on hospital problems in managing infectious patients and also the study of healthcare providers’ experiences showed that hospitals in many countries and states have many experiences and face many challenges and problems [4][5][9][10]. In addition, different hospitals and people may propose many strategies to promote the hospitals’ performance and management of infected patients [4][11][12][13][14].

Hospital management during outbreaks is different with responsibilities ranging from preventing disease spread among hospitalized patients to training the staff and hospitalized patients. In addition, during epidemics and outbreaks of communicable diseases, the capacities of hospitals to respond are different and need different management in hospitals depending on the local situations and their different requirements [15][16]. In crises, including the outbreak of the recent coronavirus, hospitals needed to acquire the capacity to confront the sudden increase in the number of referring patients requiring efficient management [17].

A review of the literature revealed that during infectious epidemics, different hospitals worldwide, especially in high-income countries, use diverse strategies to respond properly to crises [18][19][20][21][22][23].

Considering many achievements of these interventions and programs in different hospitals, around the world, in terms of readiness and responsiveness to the outbreak of infectious diseases, their evaluation can provide practical and comprehensive data for better decision-making and efficient performance at the time of epidemics. Therefore, the present scoping review aimed to evaluate hospital experiences in different countries and assess the best practices of hospitals in managing epidemic conditions. Considering mentioned conditions and due to covid 19 epidemic effects on hospital performances and high rate of mortality and the importance of gathering related practices to managing epidemic conditions, the scoping review was used as reviewing method.

## Materials and Methods

The present scoping review and content analysis was conducted in five phases in 2021. The phases included: 1: identification of the research question, 2: identification of relevant studies, 3: study selection, 4: data charting, and 5: data analysis and reporting the results. We adopted the scoping review approach from the book entitled "systematic review to support evidence-based medicine" [24]. Main keywords and sample search strategy in PubMed are shown in appendix. Also, this study’s ethics code is IR.IUMS.REC.1399.1100 of the Iran University of Medical Sciences.

Phase 1: Identification of Research Question

The primary research question was: what hospitals around the world have taken measures in managerial readiness and responsiveness to infectious epidemics, and what experiences do they have?

1.1. Inclusion and Exclusion Criteria

The papers indicating the hospitals’ measures and experiences in different countries in managerial readiness and responding to infectious epidemics in both Persian and English languages were included. The search time was unlimited.

1.2. Exclusion Criteria

Articles and reports that were not just about hospital readiness and responsiveness (studied health system or a country).

Articles quantitatively assessing a hospital’s performance or achievements.

Articles reporting a special act or the hospital’s measure as a case.

Articles only elaborated on the concepts and models of hospitals readiness or responsiveness in infectious epidemics.

Studies reporting hospitals’ readiness or responsiveness against other crises including earthquakes, floods, fires, and others.

Phase 2: Identification of Relevant Studies

We used experts’ comments, literature review, assistant librarian, EMTREE terms, and Medical Subject Headings (MeSH) to extract keywords. We collected the required data using keywords from PubMed, Scopus, Web of Sciences, and ProQuest (Appendix 1: search strategy). The search time was unlimited. We manually searched several valid journals after databases to identify and cover more papers. We conducted citation checks and reference checks for selected papers in Google Scholar. We also searched the European Association for Grey Literature Exploitation (EAGLE), Healthcare Management Information in Consortium (HMIC), and the System for Information on Grey Literature in Europe (SIGLE) for grey literature. In addition, we scrutinized the official websites of courtiers’ ministries of health and international organizations such as the World Health Organization (WHO) and the World Bank. The search strategy details are included in supplementary file 1.

Phase 3: Study Selection

**Figure-1:**
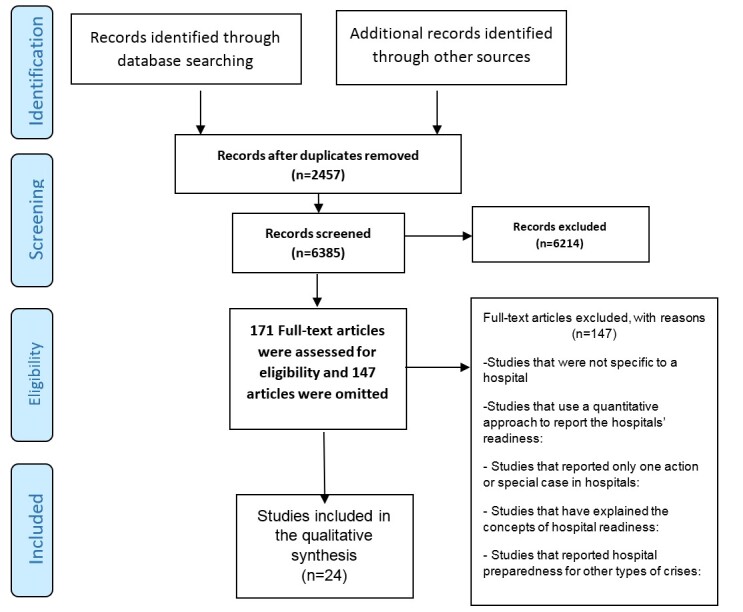
Figure1.Selection of studies on the hospital’s managerial readiness and responsiveness to infectious epidemic

Two members of the research team independently carried out the study selection. They solved the first stage of controversy through discussion. In two cases, they referred to a more experienced third party. First, they studied the titles and excluded irrelevant studies. Next, they evaluated the abstract and full texts to identify and exclude the irrelevant studies endnote (version 5) was used to organize and analyze the titles and abstracts and to detect duplication. PRISMA flowchart [25][26][27] was applied to report the results of the study selection (Figure-1). For preventing personal bias, two authors screened the papers and if in some cases did not reach an agreement, a thirdparty expert made the final decision.

Phase 4: Data Gathering

We designed two manual data charting forms using Microsoft Word (version 2016 manufactured by Microsoft company in the USA) software for data collection and consultation with research team members, according to the objectives. The first (main) form showed the general information about the article and its major results. The charted data included authors, publication year, country, article type, objectives, epidemic, major fields, subordinate fields, and conclusion. The second (complementary) form dealt with more detailed information. It avoided the perplexity of the primary form and provided more complete data about the article. This form also included the author, publication year, and main and subordinate fields. Initially, the data from three articles were extracted as a pilot study, and then, the defects in the forms were solved. Two research team members extracted data from the selected studies and solved the ambiguities.

Phase 5: Data Analysis

After collecting the data, we analyzed them through content analysis and then summarized and reported them. The content analysis identifies, analyzes, and reports patterns (themes) inside a text. It has many uses in qualitative data analysis [28][29][30][31]. Two research members independently encoded the data. The sequence of data analysis and coding was familiarization with the texts (immersion in the results), identification and extraction of primary field (identification and extraction of more relevant articles to primary fields), articles placement in their fields, revision and completion of the results of each field using the results of the articles in that field, and getting ensured about the reliability of the fields and extracted results in each field (gaining agreement between two coders through conversation and solving the problems). We reported some descriptive data using descriptive statistics, including percentage, frequency, and others. Microsoft Excel (version 2016) was used to draw charts.

## Results

Article Selection

**Figure-2:**
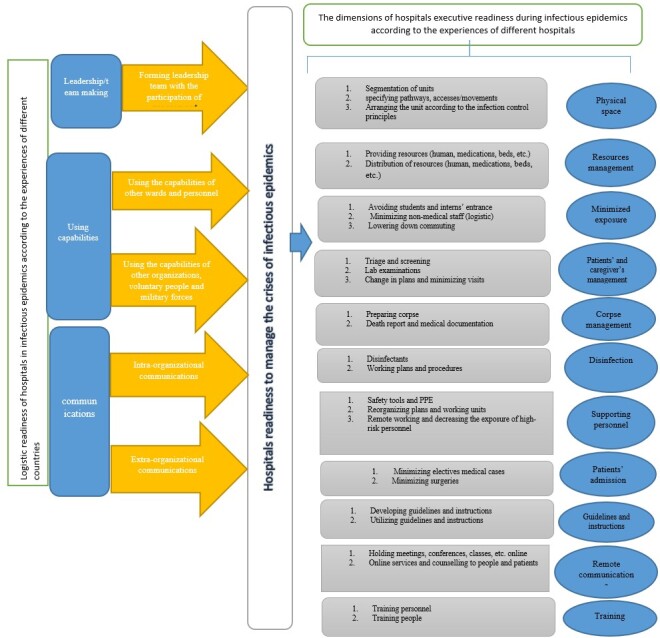
Figure2.The schematic pattern of results from the content analysis of studies on hospitals' readiness and responsiveness during infectious epidemics

We found 8842 articles from databases and other resources and excluded 2457 cases for duplication. Also, 6214 articles were excluded by title check, and 147 studies by checking their full texts. Finally, we included 24 studies (Figure-1). After a complete evaluation, we entered data charted tables (Appendix 1, provided in supplementary file 1). In this study, we identified and analyzed 24 cases of hospital experiences from 12 countries. Most were in the US (9 cases), China (3 cases), and Italy and Singapore (2 cases for each). Most of the studies (17 cases) were conducted in high-income countries and two of them were from lower middle income (Iran) and middle income (Nigeria). Covid-19 was the most reported epidemic (19 studies). Other epidemics were Flu (2 studies), Ebola (2 studies), and dengue fever (1 study). Mortality rates of infectious diseases for each country are shown in the appendix data extraction. The content analysis of measures and experiences of 24 hospitals from different countries helped classify the results under two major categories of executive readiness and logistic readiness. Executive readiness included 11 major categories and 26 sub-categories. Logistic readiness covered three major categories and five sub-categories (Figure-2, Table-1 and -2).

**Table:** Table1.Main and Sub-categories Extracted from Studies On the Executive Readiness of Hospitals during Infectious Epidemics, Explanations, Examples

**Main Category**	Sub-category	Explanation	Example (reference)
**Physical structure**	Segmentation of units	Changes in the physical space of medical units according to patients and hospital needs, dividing them into two infectious and non-infectious units	Baggiani et al (32) - Italy-in Covid-19
	specifying pathways, accesses/movements	specified commuting routes in hospital for specific people who could move under specific conditions	Macron et al (33)- Italy-in Covid-19
	Arranging the unit according to the infection control principles	special focus on the ventilation in all clinical and non-clinical units and the rooms	Buising et al. (34) -Australia-in Covid-19
**Resource management**	Providing resources (human, medications, beds, etc.)	provide necessary resources in different ways in the time of infectious epidemics	Ogoina et al (2016) (35)- Nigeria- in Covid-19 Ma J et al (2020) (36)-China-in Covid-19
	Distribution of resources (human, medications, beds, etc.).	effective and efficient distribution of resources in hospitals during epidemics	Schiller et al (2020) (37) -USA- in Covid-19
**Exposure reduction policies**	Avoiding students and interns’ entrance	Reducing exposure to disease agents and minimize the exposure to unnecessary entrance for medical students	Jebelli B, et al:2020(38)- Iran-in Covid-19
	Minimizing non-medical staff (logistic)	included remote working, homecare services, and utilizing modern technologies for visits and holding online meetings	Buising et al. (34) -Australia-in Covid-19
	Lowering down commuting	changes in commuting routes, decreased caregivers visits	Jebelli B, et al:2020(38)- Iran-in Covid-19 Buising et al. (34) -Australia-in Covid-19
**Patients and caregivers’ management **	Triage and screening	patients screening, diagnosis of infectious, separating them, and their protection	Baggiani et al. (2020) (32) - Italy-in Covid-19
	Lab examinations	nasopharyngeal swab examination and in case of apositive result the case was transferred to Covid-19 unit in emergency through specified routes	Baggiani et al. (2020) (32) - Italy-in Covid-19
	Change in plans and minimizing visits	Patients were kept and visited in isolated units in both pediatric and adult emergencies but the same was done in the pediatric clinic in the afternoons.	Cannava et al (2010) (39)-China- H1N1 influenza outbreak
**Corpse management**	Preparing corpse	Corpse transfer from the unit to the hospital morgue must use proper Personal Protection Equipment (PPE). The corpses should be covered properly in a bag. After they enter the morgue they must be kept in a cool area preferably in the lower row of the shelf. Also it must be written on a black board that this body is the body of covid-19 patient. Before the coffin is closed, a member of the family must be allowed to see the body at least from a distance of 2 meters	Baggiani et al. (2020) (32) Italy-in Covid-19
	Death report and medical documentation	Deaths from the disease in hospitals were assessed by a group of specialists from different fields, infection control officer, and a trained nurse and they have prepared report and sent it to the hospital and local authorities	Arya SC, et al:2004 (40)-India- dengue outbreak
**Disinfection plans**	Disinfectants	Using Ethyl alcohol biocides (70%), Hydrogen peroxide (0.5%), Sodium hypochlorite (0.1-0.5 chlorine free) and other sanitizers to disinfect medical equipment according to the European standard (476,EN 14).	Baggiani et al (2020) (32) Italy-in Covid-19
	Working plans and procedures	sanitizing staff were properly trained and they were cleaning Covid-19 patients’ rooms	Jebbeli et al. (2020) (38) Iran-in Covid-19 Baggiani et al (2020) (32) Italy-in Covid-19
**Staff support**	Safety tools and PPE	Providing safety personal protection equipment’s including mask, shield, and etc.	Walsh A,2021 (41), Canada-in Covid-19 Wong et al (2020) (42), Singapore-in Covid-19
	Reorganizing plans and working units	supportive interventions, team working, and self-care, flexibility, and tolerance techniques were provided by hospitals managers to health staff	Gupta S and Federman DG.2020 (43) - USA-in Covid-19
	Remote working and decreasing the exposure of high-risk personnel	sending high-risk staff for remote work and In some cases, these people were isolated in their homes for 12 weeks	Britton CR,2020 (44) - UK-in Covid-19
	Changing Patient Admission Policies	Minimizing electives medical cases	Minimizing elective and non-emergency patients’ admission. Usually, it happens for minimizing the probability of patients and their caregivers’ infection, decreasing medical equipment consumption and allocating them to infectious patients, and concentrating health staff on infectious patients	Jebelli B, et al:2020(38)- Iran-in Covid-19
	Minimizing surgeries	lowering the number of elective surgeries and the allocation of almost 80% of Whole Time Equivalents (WTE) to Covid-19 patients by changing working schedules and concentration of health staff on Covid19 patients.	Britton CR,2020 (44) - UK-in Covid-19 Iannuzzi NP, et al:2020(45) - USA-in Covid-19
	Instructions and Guidelines	Developing guidelines and instructions	Designing and developing guidelines and protocols to treat infected patients	Filice et al (2013) (46)-USA- H1N1 influenza outbreak Lombardi et al. (2020) (47) - USA-in Covid-19
	Utilizing guidelines and instructions	using national guidelines and protocols and developing protocols based on their needs	Buising et al (2021) (34) Australia-in Covid-19
	Using telecommunications	Holding meetings, conferences, classes, etc. online	Using remote communications and cyberspace for organizational goals during epidemics	Shao et al. (2020) (48)-China- in Covid-19 Jebbeli et al. (2020) (38) Iran-in Covid-19
	Online services and counselling to people and patients	providing patients and community online services on medical and mental health	Shao et al. (2020) (48)-China- in Covid-19 Schwaezkopf et al (2020) (49) USA-in Covid-19
**Training**	Training personnel	Training personnel about self-protection, using PPE, ways of transmission, etc. during epidemics	Chopra V, et al:2020 (50)- USA-in Covid-19 Xiang et al (2020) (51) -China- in Covid-19
	Training people	general training of patients about self-protection	Xiang et al (2020) (51) -China- in Covid-19

**Table:** Table2: main and sub-categories extracted from studies on the logistic readiness of hospitals during infectious epidemics, explanations, examples

**Main category**	Sub-category	explanation	Example (reference)
**Leadership/team making**	**Forming leadership team with the participation of managers and different stakeholders **	Some hospitals at the onset of epidemic formed a leadership team with the presence of different specialists and different people from different units	Gupta S and Federman DG.2020 (44) - USA-in Covid-19 Walsh A,2021 (41), Canada-in Covid-19
**Utilizing capabilities**	**Utilizing the capabilities of other wards and specialties **	During an outbreak some wards such as emergency and ICUs are more engaged than others. Due to the decreased number of patients there must be a plan to utilize the staff and facilities of other wards.	Iannuzzi NP, et al:2020(45) - USA-in Covid-19 Britton CR,2020 (43) - UK-in Covid-19
	**Using the capabilities of other governmental organizations, military forces, voluntary people and NGOs .etc. **	Many hospitals have plans to use the capabilities of organizations, military forces, voluntary people and NGOs along with their capabilities in other wards. They were successful in utilizing them especially in terms of resources.	Jebelli B, et al:2020(38)- Iran-in Covid-19 Lateef O, et al:2015 (52)-USA- Ebola virus disease (EVD)
**communications**	**Intra-organizational**	Effective communication with other wards and units inside hospitals and other hospitals and other parts of health system to fulfill needs and defects was the routine plan and priority of hospitals during outbreak	Britton CR,2020 (43) - UK-in Covid-19 Marcon E, et al;2020 (33) - Italy-in Covid-19 Buising KL, et al:2021 (34)-Australia-in Covid-19
**Inter-organizational**	Many hospitals, along with their intra-organizational communications, plan to establish extra-organizational communications with other organizations and layers of community to advance their plans and control the outbreak efficiently	Chopra V, et al:2020 (50)- USA-in Covid-19 Arya SC, et al:2004 (40)-India- dengue outbreak

Executive Readiness: Dimensions

The hospital executive readiness included 11 main categories (Physical structure/Resource management/Exposure reduction policies /Patient and caregivers’ management/Corpse management/Disinfection plans/Staff support/Changing Patient Admission Policies/ Instructions and Guidelines/Instructions and Guidelines/Using telecommunications/Training) and 26 subcategories during infectious outbreaks. Table-1 shows their description and examples.

Logistic Readiness: Dimensions

The results of the major categories (leadership/team making, communication, and using capabilities) and five subcategories in logistic readiness of hospitals during infectious outbreaks and their explanation and examples are presented in Table-2.

## Discussion

By reviewing and content analyzing the experience and behavior of 24 hospitals in different countries during infectious epidemics (especially Covid-19), we divided their readiness and responsiveness into two main dimensions of executive readiness (11 categories and 26 subcategories) and logistic readiness (3 categories and 5 subcategories). This study included 14 categories and 31 subcategories.

Evaluation of studies on hospitals’ readiness and responsiveness during infectious epidemics showed their comprehensiveness and maximum conceptual coverage. For example, Ippolito et al. (2006), preliminarily reviewing the literature on hospitals readiness and responsiveness during infectious epidemics and bioterrorism, pointed out five categories, including clinical awareness and education, initial investigation and management, surge capacity, communication, and caring for staff and others [53].

In a comprehensive report by WHO (2014) on the readiness of hospitals during infectious epidemics, the authors mentioned efficient management, an infection control plan, communications, human resource management, logistics, hospital pharmacy, hospital emergency, hospital laboratory, providing basic services, stability of services, mental-psychological services, supports, and capacity of surgery rooms as the most crucial factors [54].

Ghotbi et al. (2020) evaluated WHO reports and scientific papers and suggested hospital management strategies during Covid-19. They discussed triage management, acute respiratory disease clinics, patients’ quarantine in healthcare-providing centers, procedures and experiments, staff, call tracking, the healthcare system, and general education (non-pharmacological interventions) [55].

Considering infectious diseases-related crisis involving a large number of people in a community and high hospital occupancy, and a high potential for exposure of hospital managers and staff to infectious agents, hospitals should be highly prepared and increase their capabilities to respond to patients in the short term. Therefore, adequate resources, instructions, and evidence can help hospital managers. The model presented in this study and the dimensions derived from a review of different hospital experiences during the epidemic can be used as a practical and comprehensive guide by hospital and health system administrators. The key points are differences in hospital needs, regional characteristics, hospital readiness and responsiveness, depending on the country or region. Therefore, each hospital should plan to use models and instructions according to the local setting and its circumstances.

A key issue highlighted by the current study and research reviewed in other resources is adequate staff support and management. A comprehensive plan is needed to support health workers in many areas before, during and after a crisis to meet needs at work, at home, and in the community. A major challenge for the healthcare system is the lack of human resources, which must be adequately addressed before a crisis occurs [56)] because manpower is a very critical factor in managing crisis epidemics, supporting them should be considered.

The US has increased inter-sectorial collaboration among the responsible organization to employ a new working force, facilitated foreign human working forces, and avoided the migration of health staff during Covid-19 [57]. Healthcare providers are exposed to infection. Therefore, they should have access to PPE and their respective user guides [58]. In line with the former studies, the present study highlighted the application of PPE, including gowns, gloves, masks, and face shields [59][60]. During pandemics, training and using scientific evidence is necessary to promote the efficiency of the health system and health staff durability [61]. Another challenge is health staff psychological problems caused by the pandemic crucial conditions, increased number of patients, patient’s clinical condition, specialty services, heavy workload, exhaustion from long working shifts, fear of transmitting the infection to self or family, patients and coworkers’ death, insufficient PPE, following health protocols at work and at home, lack of trust and support, social distancing policies, and long-term separation from family and children [5][6][11][62][63][64]. Fear of transmitting the infection to the family is a big problem for medical staff, caused by close contact with patients.

Due to the long-term commune of the infectious diseases and delayed symptoms, they fear they might transmit the disease to their family members. This challenge can be solved by proper management of personnel working schedules and regular shifts and resting intervals, employing active and new working force, regular medical examinations to be ensured their health, especially before their offs, and supporting and caring for medical staff, psychological, and self-care facilities and services [4][6][65][66].

The literature review of hospital experiences shows that hospitals face many administrative challenges during the first days of an infectious disease outbreak (especially Covid-19). Challenges facing hospitals include rapid triage of patients, segregation of suspected patients and assignment of levels of care, lack of support from medical staff, and challenges in communication and collaboration in multidisciplinary teams.

Managers can control the crises by a set of measures, including long-term planning before crises, forming crisis teams during the crises and assigning precise roles to the members, holding regular meetings, media conferences, using communication tools to train and avoid rumors, precise supervision on infection prevention and control, following health protocols and punishing those who violate the protocols, supporting medical staff and having the humane attitude, sending high-risk staff to remote work, providing insurance coverage to working damages, providing staff with safety, accommodation, welfare, and psychological facilities, and forming a professional team to train new staff.

Pandemics and epidemics pose a variety of challenges and problems to health-providing systems and disrupt routine situations and their control [4, 11, 13, 67). Therefore, managers can effectively respond to similar future situations by using their experience and consulting with colleagues in other countries and international organizations.

The present study showed that most of the reported experiences came from high-income countries. Because of the differences in the social, economic political situations, and health systems of these countries with low and middle-income countries (LMICs), we should consider the situations of each country and even province in modeling the policies and experiences and make necessary changes to adopt the policies with indigenous situations. The present review comprehensively and extensively collects and analyzes the evidence and experiences of hospitals on their readiness and responsiveness against infectious epidemics and provides practical and comprehensive data about the readers and policymakers. It faces several limitations, including limited access to the evidence and experiences of other countries.

The probable reason can be the lack of published experiences in other countries and/or their publication in local (non-English) languages. Also, because the authors can read only English and Persian articles, only these article results are mentioned in this study.

## Conclusion

The crises caused by infectious outbreaks have affected an extensive part of the population and community, cause many patients to refer to the hospitals, and threaten the medical personnel and managers.

These ensure that hospitals are prepared to the highest level and improve their ability to effectively respond to patient needs and referrals in the short term. Therefore, the presence of resources, instructions, and enough evidence can greatly assist hospital managers and authorities.

In this review, we analyzed 24 different hospitals’ experiences and measures from diverse countries during infectious outbreaks (especially Covid-19). We highlighted dimensions of their managerial readiness and responsiveness during infectious outbreaks and presented them in two major categories of executive readiness and logistic readiness. We believe this study can be a practical and comprehensive guide for hospital and health system managers and authorities.

## Acknowledgements

This study was funded by the Iran University of Medical Sciences as a part of PhD thesis in health care management by grant number 99-3-37-19584.

## Conflict of Interest

There is no competing interest.
